# Evaluation of chronic orofacial pain and its relationship to the microvascular facial response in women with fibromyalgia

**DOI:** 10.1007/s10067-025-07570-1

**Published:** 2025-07-30

**Authors:** María del Carmen Villaverde-Rodríguez, María Correa-Rodríguez, Antonio Casas-Barragán, Rosa María Tapia-Haro, María Encarnación Aguilar-Ferrándiz

**Affiliations:** 1 PhD Biomedicine Program, Faculty of Health Sciences (Granada), University of Granada, Ave. de La Ilustración, 60, 18016 Granada, Spain; 2 Department of Nursing, Faculty of Health Sciences (Granada), University of Granada, Ave. de La Ilustración, 60, 18016 Granada, Spain; 3 Department of Physiotherapy, Faculty of Health Sciences (Granada), University of Granada, Ave. de La Ilustración, 60, 18016 Granada, Spain; 4 Instituto de Investigación Biosanitaria ibs.GRANADA, Granada, Spain

**Keywords:** Fibromyalgia, Orofacial pain, Quality of life, Thermography

## Abstract

**Background:**

Recent investigations suggest that central nervous system dysfunction and changes in peripheral blood microcirculation may explain the symptoms of fibromyalgia syndrome (FMS).

**Objectives:**

To analyze the presence of chronic orofacial pain (COP) and assess facial skin microcirculation through surface temperature in women with FMS and healthy controls and to examine the relationship between peripheral facial skin temperature and oral symptomatology in FMS.

**Design and methods:**

An observational case–control study was conducted with 46 women with FMS and 44 healthy women. Infrared thermography was used to assess peripheral facial skin temperature, and facial pressure pain thresholds (PPTs), mouth opening measurement, the Craniofacial Pain and Disability Inventory (CF-PDI), and the Orofacial Visual Analog Scale (VAS) were used to assess COP.

**Results:**

Significant differences were found in orofacial-VAS (*p* ≤ 0.001), orofacial-PPTs (*p* ≤ 0.001), mouth opening (*p* = 0.003), and CF-PDI (*p* ≤ 0.001) between women with FMS and controls. No significant differences were identified in peripheral facial skin temperature between cases and controls, but significant associations were observed between facial thermography and oral symptomatology.

**Conclusion:**

Women with FMS exhibited greater orofacial pain and worse orofacial quality of life than healthy women, with associations between facial temperature and oral symptoms.

**Table:** 

**Key Points** • *Women with fibromyalgia syndrome present chronic orofacial pain.* • *Mouth opening limitation and lower orofacial pressure pain threshold are observed.* • *The presence of orofacial pain has a negative impact on quality of life.* • *An association between facial thermography and oral pain symptoms is observed.*

## Introduction

Fibromyalgia syndrome (FMS) is a multisymptomatic condition characterized by the presence of widespread chronic musculoskeletal pain with low pain tolerance, hyperalgesia, and allodynia [1]. FMS symptoms include somatic manifestations such as headaches, orofacial pain, and swelling [2] as well as cognitive disturbances like sleep problems, anxiety, and irritability [3]. Furthermore, FMS has a considerable impact on the quality of life (QoL) and level of disability of patients [4]. The worldwide prevalence of the FMS is 2.1%, and in Europe, it is 2.31%, and it is more likely in women than men (4.49% versus 0.2%; ratio 9:1) [5]. The etiopathogenesis of FMS is still unknown, but it has been postulated that the central nervous system (CNS) might play a relevant role in diffuse musculoskeletal pain since it may be related to central neural mediation that alters sensory processing and pain perception [6].

Previous studies indicate heterogeneity in the clinical symptomatology of chronic orofacial pain (COP) in FMS [7]. COP must be present for more than 4 months and may include toothache, temporomandibular dysfunction (TMD), and others [8, 9]. The etiology of TMD is multifactorial and is due to functional, structural, and psychological factors [10]. The prevalence of TMD-related oral symptoms ranges from 59.3 to 80.6% in FMS [11]. Although the link between FMS and TMD remains unclear, widespread musculoskeletal pain in FMS may contribute to stomatognathic system imbalance [10]. A parameter associated with pain is hyperthermia, defined as an increase in temperature related to a local vasodilation response [8]. However, hyperthermia associated with orofacial pain has been poorly investigated and vaguely described [12]. A recent hypothesis has linked pain to microvascular dysfunction. Albrecht et al. [13] determined the existence of peripheral neuropathology between cutaneous arterioles and arteriole-venule shunts (AVS) in patients with FMS. The AVS have greater innervation in patients with FMS, with a greater presence of vasodilatory sensory fibbers. These fibbers play an important role in chronic pain and sensitization conditions due to the small-caliber sensory innervation of the cutaneous vasculature and thermoregulation [13].

To date, contradictory data has been published regarding the potential association between FMS and orofacial pain. Moreover, no studies have assessed the peripheral blood flow of the skin of the face throughout thermography and its association with facial pain intensity, tenderness, mouth opening, and orofacial quality of life. In this context, we hypothesized that women with FMS report more COP compared to healthy controls, along with impaired peripheral blood flow of the face and poorer QoL. Thus, the aims of this study were (I) to compare the orofacial pain intensity, facial pressure pain threshold, mouth opening, orofacial QoL and facial skin surface temperature in women with FMS and healthy controls and (II) to analyze the relationship between peripheral temperature of the facial skin and oral symptomatology, including facial pain intensity, facial PPTs, mouth opening, and orofacial QoL in women with FMS.

## Materials and methods

### Study design and participants

An observational case–control study was conducted between May and October 2019 involving women diagnosed with FMS. Participants were recruited through the Fibromyalgia Association of Granada (AGRAFIM, Spain), which facilitated contact with women who voluntarily agreed to participate. The fibromyalgia association of Granada (Spain; AGRAFIM) facilitated the recruitment of women with FMS who voluntarily wanted to participate in this study. A total of 90 women between 30 and 70 years of age participated in the study, including 46 women diagnosed with FMS and 44 healthy controls. The selection of a total of 46 women diagnosed with FMS was based on availability and feasibility during the recruitment period, as well as on sample sizes similar to those reported in related previous studies [8, 14, 15]. The control group was composed of relatives or friends of FMS participants and volunteers recruited through advertisements at the University of Granada.

Inclusion criteria for the FMS group were a clinical diagnosis of FMS confirmed by a rheumatologist within the Andalusian Public Healthcare System (Spain) and the American College of Rheumatology criteria for FMS [2, 16]. Exclusion criteria were male sex, presence of cardiac, renal, or hepatic insufficiency, previous rheumatic disease, severe physical disability, fever after infection in the last 2 weeks, hypotension/hypertension, psychiatric disease, neurological disorders, cancer, history of surgery, treatment with vasoactive or anticoagulant drugs or history of drugs, skin alterations, and presence of TMD, performed with the biobehavioural model of pain, which includes physical signs and symptoms (axis I) and psychological signs and symptoms and disability factors (axis II) [17]. Healthy women had no diagnosis of FMS and met the same exclusion criteria as the FMS group.

During the visit, informed consent was obtained, the objective of the study was explained, and any questions were answered. The study was approved by the Ethics Committee of the University of Granada Spain (Approval number, 718-N-18). This research was performed in strict compliance with the international code of medical ethics established by the World Medical Association and the Declaration of Helsinki. Participants completed structured questionnaires regarding their age, weight, height, medical history, including mouth infections, presence of orthodontics and implants, medications, age of fibromyalgia diagnosis, and menopause status. Body mass index (BMI) was calculated as weight (kg)/height (m^2^).

### Peripheral vascular blood flow

**Fig. 1:**
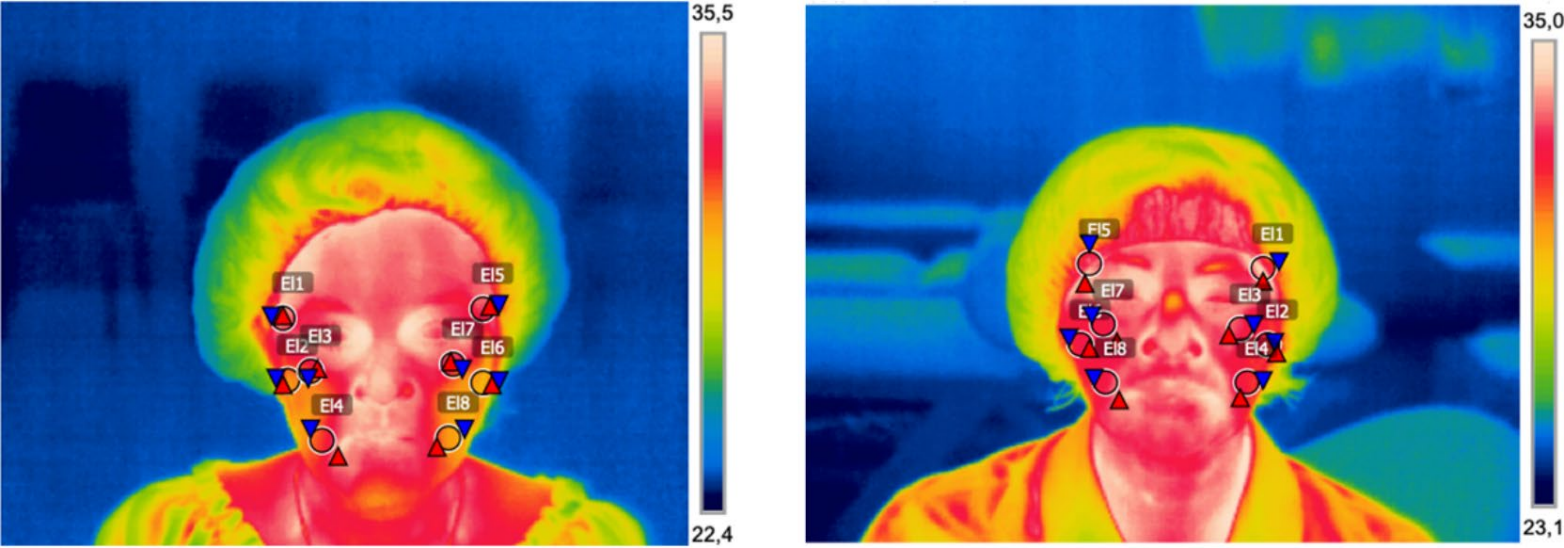
Thermography image of ROI facials of a woman diagnosed with fibromyalgia and healthy control

The peripheral temperature of the facial skin was assessed by an infrared thermography camera (FLIR B335, FLIR Systems AB, Täby, Sweden). The atmospheric temperature of the camera was set at 20 °C, and the spectral emissivity was set at 0.98 [18, 19]. All thermograms were conducted in compliance with the recommendations proposed by the European Association of Thermography and American Academy of Thermology [20, 21], and they were carried out by the same specialist. The participants stayed in a sitting position in a quiet room at a constant room temperature of 20 °C following an acclimatization period of 20 min. The tripod was placed 0.71 m from the participant [18]. It was verified that the facial area to be photographed was free of makeup, accessories, and hair. The participants were installed on a stool in a straight position, with their feet on the floor and their gaze fixed on one point. The thermal camera was programmed to capture the regions of interest (ROI) chosen for the facial analysis [22]. The photographs covered the following ROI: temporomandibular joint frontal (TMJ) area, located anterior to the tragus and centred over the condylar region, proximal masseter frontal (PM) region, corresponding to the upper third of the masseter muscle, just below the zygomatic arch, distal temporal frontal (DT) region, located in the lateral frontal aspect of the temporal muscle; and distal masseter frontal (DM) region, located over the lower third of the masseter muscle near the mandibular angle (Fig. 1) [18, 22].

Frontal photographs were obtained and analyzed. The images obtained were analyzed using the FLIR ResearchIR Thermographic Image Analysis Software (FLIR Systems, Inc.). The temperature scale was adjusted to the thermal content of the image [19]. The images obtained were analyzed through a circular area with a diameter of 11 × 11 mm (mm), obtaining the minimum, maximum, and average temperature of the selected ROI [22].

### Core body temperature

Core body temperature was assessed in the external auditory canal using an infrared thermometer (Infrared Dermal Thermometers, Exergen). This non-invasive method reflects an accurate measurement of core temperature due to the association of the tympanic artery with the hypothalamus, which is the thermoregulatory center that regulates the homeostatic control of body temperature [23]. The temperature of the region was also recorded axillary. We performed three measurements in the ear and armpit on the participant’s dominant side.

### Facial pressure pain thresholds (PPTs)

A digital pressure algometer (Wagner Instruments, Greenwich, USA) was used to measure facial pressure pain thresholds (PPTs). PPTs were assessed bilaterally over the anatomical points: (1) temporomandibular joint left and right (TML, TMR): located anterior to the tragus of the ear, in the region where the mandibular condyle articulates with the mandibular fossa of the temporal bone; (2) proximal temporal left and right (PTL, PTR): located 3 cm (cm) above the line drawn between the upper edge of the ear and the external corner of the eye; (3) distal temporal left and right (DTL, DTR): located 1 cm cranial and anterior to the external edge of the ear; (4) masseter proximal left and right (MPL, MPR): located 2.5 cm anterior to the tragus; and (5) masseter distal left and right (MDL, MDR): located 1.5 cm caudal from the anterior point and 2 cm anterior to the angle of the jaw [24]. The mean value of three consecutive trials was calculated and used for the main analysis. A 30-s resting interval was allowed between each measurement [24].

### Visual Analog Scale Orofacial (VAS)

To assess the subjective intensity of orofacial pain, we used a VAS [25]. Patients were asked to indicate the intensity of their orofacial pain on a 100 mm horizontal line. The VAS for orofacial pain is a point numeric rating scale, with 0 representing “no pain” and “unbearable pain.” The patients were asked to assign a number to their average pain in the last week. VAS scores 0–4 mm: no pain, 5–44 mm: mild pain, 45–74 mm: moderate pain, and 75–100 mm: severe pain [25].

### Mouth opening

The distance was measured with a tape measure in mm between the upper and lower central incisors to assess whether there may be an alteration in the opening of the mouth, considering a normal opening between 35 and 44 mm, hypomobility < 35 mm, and hypermobility > 44 mm. The participants were placed on a stretcher in a supine position and told to open their mouths as wide as possible without pain [26].

### Orofacial quality of life

The Craniofacial Pain and Disability Inventory (CF-PDI) was used to assess the functional level and the degree of orofacial pain and disability. This self-questionnaire consists of 21 items that assess craniofacial pain and disability (14 items) (CF-PDI pain and disability) and the functional status of the jaw (7 items) (CF-PDI jaw functional status), with four possible Likert-type responses, scoring from 1 (no problem) to 4 (maximum problem). The higher the score, the greater the condition of the orofacial pain [27].

### Statistical analysis

Data were analyzed with SPSS© version 22.0 (IBM Corporation, Armonk, NY, USA). The Kolmogorov–Smirnov test was used to analyze the normality of the distribution of the variables (*p* > 0.005). Due to their skewed distribution, height, axillary temperature, tympanic temperature, VAS orofacial pain, PPTs, TMD temperature, mouth opening, and CF-PDI-pain variables were log-transformed before being included in the models. To aid interpretation, the data were back-transformed from the log scale for presentation in the results. To compare the two groups, we used the Mann–Whitney *U* test and Student’s *t*-test for continuous data and *X*^2^ for categorical data. Differences in clinical variables between women with FMS and healthy women were determined using analysis of covariance (ANCOVA) after adjusting for age, BMI, and menopause status. Linear regression analyses were conducted to determine the association between peripheral temperature at the skin surface of the face and oral symptomatology, including facial pain intensity, facial PPTs, mouth opening, and orofacial QoL, after adjusting for age, BMI, and menopause status. The results are reported as a percentage change (*β*) with 95% confidence intervals (95% CI). *p*-values of < 0.005 were considered to be statistically significant.

## Results

### Demographic and clinical data

Women with FMS presented significantly higher levels of VAS, reduced PPTs, and decreased mouth opening compared to controls. QoL was also impaired (CF-PDI scores). No significant differences were found in overall facial skin temperature, though specific associations between thermographic ROIs and clinical variables were observed. Detailed values are provided in Tables 1, 2, 3, 4, and 5.

**Table 1:** Demographic and clinical variables of women with fibromyalgia and healthy controls

	Cases (*N* = 46)	Controls (*N* = 44)		
	Mean; SD		Mean; SD	*p* value
**Demographic characteristics**				
Age (years)	56.45; 6.93	54.95; 8.11		0.170
Height (cm)	1.59; 0.05	160.78; 7.59		0.252
Weight (kg)	71.90; 15.54	66.90; 8.97		0.207
BMI (kg/m^2^)	28.96; 7.56	26.23; 3.60		0.206
Status menopause	29 (59.6)	17 (38.63)		0.005^a^
Axillary temperature (°C)	35.37; 0.98	34.75; 0.98		0.005^a^
Tympanic temperature (^°^C)	35.82; 0.80	35.79; 0.66		0.813
**Medications**				
Analgesics	22 (47.82)	2 (4.54)		< 0.001^a^
Anti-inflammatory	10 (21.73)	1 (2.27)		< 0.001^a^
Anxiolytics	9 (19.56)	1 (2.27)		< 0.001^a^
Antidepressive	14 (30.43)	1 (2.27)		< 0.001^a^
**Clinical data**				
VAS orofacial pain (mm)	55.96; 30.89	5.10; 13.53		< 0.001^a^
PPTs				
DTR	0.698; 0.37	2.00; 0.99		< 0.001^a^
DTL	0.72; 0.43	2.18; 0.90		< 0.001^a^
PTR	0.50; 0.26	1.66; 0.77		< 0.001^a^
PTL	0.55; 0.35	1.63; 0.73		< 0.001^a^
MDR	0.45; 0.24	1.17; 0.55		< 0.001^a^
MDL	0.43; 0.29	1.21; 0.51		< 0.001^a^
MPR	0.49; 0.28	1.34; 0.59		< 0.001^a^
MPL	0.54; 0.40	1.32; 0.69		< 0.001^a^
TMR	0.56; 0.29	1.59; 0.69		< 0.001^a^
TML	0.57; 0.32	1.60; 0.69		< 0.001^a^
Facial thermography				
TMJ T°	31.99; 1.02	31.85; 1.73		0.656
DT T°	37.36; 24.60	33.31; 1.22		0.301
PM T°	33.10; 1.06	32.96; 1.24		0.588
DM T°	32.87; 1.09	33.02; 1.30		0.588
Mouth opening (mm)	38.47; 14.05	48.55; 17.81		0.003^a^
CF-PDI total	37.08; 14.37	5.20; 5.57		< 0.001^a^
CF-PDI pain	28.40; 10.53	4.15; 4.29		< 0.001^a^
CF-PDI jaw functional	8.68; 5.16	1.05; 1.90		< 0.001^a^

Data are expressed as mean and range or frequency and percentage. Student’s *t* or Mann–Whitney *U* tests (continuous variables) and chi squared tests (categorical variables)

*SD* standard deviation, *VAS* Visual Analog Scale Orofacial, *PPTs* Pressure Pain thresholds, *DTR* distal temporal right, *DTL* distal temporal left, *PTR* proximal temporal right, *PTL* proximal temporal left, *MDR* masseter distal right, *MDL* masseter distal left, *MPR* masseter proximal right, *MPL* masseter proximal left, *TMR* temporomandibular joint right, *TML* temporomandibular joint left, *DT T°* distal temporal frontal mean temperature, *TMJ T°* temporomandibular joint frontal mean temperature, *PM T°* proximal masseter frontal mean temperature, *DM T°* distal masseter frontal mean temperature, *CF-PDI* Craniofacial Pain and Disability Inventory total, *CF-PDI pain* Craniofacial Pain and Disability Inventory functional pain and disability, *CF-PDI functional* Craniofacial Pain and Disability Inventory

^a^Analysis of covariance (ANCOVA) after adjusting for age, BMI, and menopause status

**Table 2:** Association between peripheral temperature of the distal temporal frontal assessed by an infrared thermography camera and facial pain in women with FMS

*Distal temporal frontal mean temperature*			
	Cases (*N* = 46)		
	*β*	95% CI	*p* value
VAS orofacial pain (mm)	0.119	(− 0.193, 0.389)	0.498
PPTs			
DTR	− 0.177	(− 23.529, 8.183)	0.333
DTL	− 0.326	(− 26.128, 0.739)	0.063
PTR	− 0.143	(− 22.299, 9.490)	0.419
PTL	− 0.174	(− 36.071, 39.515)	0.300
MDR	− 0.290	(− 29.022, 3.453)	0.119
MDL	− 0.239	(− 22.710, 2.702)	0.119
MPR	− 0.209	(− 29.022, 3.453)	0.119
MPL	− 0.363	(− 28.511, 0.611)	0.041^a^
TMR	− 0.162	(− 22.535, 8.359)	0.358
TML	− 0.285	(− 26.287, 2.600)	0.105
Mouth opening (mm)	− 0.038	(− 0.745, 0.605)	0.835
CF-PDI total	0.893	(− 0.467, 0.750)	0.640
CF-PDI pain	0.126	(− 0.529, 1.120)	0.085
CF-PDI jaw functional	− 0.028	(− 1.778, 1.517)	0.873

*Beta (β)* regression coefficient, adjusted for age, menopause status, and body mass index, *95% CI* confidence interval, *VAS* Visual Analog Scale Orofacial, *PPTs* Pressure Pain thresholds, *DTR* distal temporal right, *DTL* distal temporal left, *PTR* proximal temporal right, *PTL* proximal temporal left, *MDR* masseter distal right, *MDL* masseter distal left, *MPR* masseter proximal right, *MPL* masseter proximal left, *TMR* temporomandibular joint right, *TML* temporomandibular joint left, *CF-PDI* Craniofacial Pain and Disability Inventory total, *CFPDI* pain Craniofacial Pain and Disability Inventory functional pain and disability, *CF-PDI* functional Craniofacial Pain and Disability Inventory functional status of the jaw

^a^Significance level *p*

**Table 3:** Association between peripheral temperature of the temporomandibular joint assessed by an infrared thermography camera and facial pain in women with FMS

*Temporomandibular joint frontal mean temperature*			
	Cases (*N* = 46)		
	*β*	95% CI	*p* value
VAS orofacial pain (mm)	0.095	(− 0.009, 0.015)	0.591
PPTs			
DTR	− 0.116	(− 0.874, 0.456)	0.527
DTL	− 0.114	(− 0.768, 0.401)	0.528
PTR	− 0.158	(− 0.953, 0.367)	0.373
PTL	− 0.138	(− 1.401, 0.588)	0.413
MDR	− 0.424	(− 1.425, − 0.131)	0.020^a^
MDL	− 0.144	(− 0.762, 0.323)	0.416
MPR	− 0.424	(− 1.425, − 0.131)	0.020^a^
MPL	− 0.257	(− 1.028, 0.170)	0.155
TMR	− 0.145	(− 0.908, 0.381)	0.413
TML	− 0.169	(− 0.908, 0.324)	0.343
Mouth opening (mm)	− 0.077	(− 0.034, 0.022)	0.677
CF-PDI total	0.319	(− 0.001, 0.047)	0.065
CF-PDI pain	0.316	(− 0.002, 0.64)	0.067
CF-PDI jaw functional	0.230	(− 0.022, 0.112)	0.182

*Beta (β)* regression coefficient, adjusted for age, menopause status, and body mass index, *95% CI* confidence interval, *VAS* Visual Analog Scale Orofacial, *PPTs* Pressure Pain thresholds, *DTR* distal temporal right, *DTL* distal temporal left, *PTR* proximal temporal right, *PTL* proximal temporal left, *MDR* masseter distal right, *MDL* masseter distal left, *MPR* masseter proximal right, *MPL* masseter proximal left, *TMR* temporomandibular joint right, *TML* temporomandibular joint left, *CF-PDI* Craniofacial Pain and Disability Inventory total, *CF-PDI pain* Craniofacial Pain and Disability Inventory functional pain and disability, *CF-PDI functional* Craniofacial Pain and Disability Inventory functional status of the jaw

^a^Significance level *p* < 0.05

**Table 4:** Association between peripheral temperature of the proximal masseter frontal assessed by an infrared thermography camera and facial pain in women with FMS

*Proximal masseter frontal mean temperature*			
	Cases (*N* = 46)		
	*β*	95% CI	*p* value
VAS orofacial pain (mm)	0.126	(− 0.008, 0.017)	0.470
PPTs			
DTR	0.113	(− 0.477, 0.900)	0.536
DTL	0.007	(− 0.596, 0.620)	0.969
PTR	0.024	(− 0.644, 0.735)	0.894
PTL	0.038	(− 0.022, 1.155)	0.821
MDR	− 0.242	(− 1.169, 0.245)	0.193
MDL	0.040	(− 0.503, 0.630)	0.820
MPR	− 0.242	(− 1.169, 0.245)	0.193
MPL	− 0.042	(− 0.711, 0.564)	0.817
TMR	− 0.010	(− 0.692, 0.653)	0.954
TML	− 0.032	(− 0.805, 1.895)	0.418
Mouth opening (mm)	0.145	(− 0.017,0.040)	0.426
CF-PDI total	0.302	(− 0.003, 0.048)	0.079
CF-PDI pain	0.243	(− 0.010, 0.059)	0.159
CF-PDI jaw functional	0.327	(− 0.001, 0.134)	0.053

*Beta (β)* regression coefficient, adjusted for age, menopause status, and body mass index, *95% CI* confidence interval, *VAS* Visual Analog Scale Orofacial, *PPTs* Pressure Pain thresholds, *DTR* distal temporal right, *DTL* distal temporal left, *PTR* proximal temporal right, *PTL* proximal temporal left, *MDR* masseter distal right, *MDL* masseter distal left, *MPR* masseter proximal right, *MPL* masseter proximal left, *TMR* temporomandibular joint right, *TML* temporomandibular joint left, CF-PDI Craniofacial Pain and Disability Inventory total, *CF-PDI pain* Craniofacial Pain and Disability Inventory functional pain and disability, *CF-PDI functional* Craniofacial Pain and Disability Inventory functional status of the jaw

**Table 5:** Association between peripheral temperature of the distal masseter frontal assessed by an infrared thermography camera and facial pain in women with FMS

*Distal masseter frontal mean temperature*			
	Cases (*N* = 46)		
	*β*	95% CI	*p* value
VAS orofacial pain (mm)	0.041	(− 0.029, 0.053)	0.556
PPTs			
DTR	− 0.017	(− 0.028, 0.051)	0.558
DTL	− 0.094	(− 0.024, 0.014)	0.602
PTR	− 0.196	(− 0.033, 0.009)	0.269
PTL	− 0.095	(− 0.041, 0.023)	0.574
MDR	− 0.357	(− 0.043, 0.000)	0.053
MDL	− 0.171	(− 0.026, 0.009)	0.334
MPR	− 0.357	(− 0.043, 0.000)	0.053
MPL	− 0.173	(− 0.029, 0.010)	0.341
TMR	− 0.277	(− 0.026, 0.004)	0.113
TML	− 0.090	(− 0.025, 0.015)	0.616
Mouth opening (mm)	− 0.064	(− 0.001, 0.001)	0.727
CF-PDI total	0.415	(0.000, 0.002)	0.015^a^
CF-PDI pain	0.384	(0.000, 0.002)	0.024^a^
CF-PDI jaw functional	0.350	(0.000, 0.004)	0.039^a^

*Beta (β)* regression coefficient, adjusted for age, menopause status, and body mass index, *95% CI* confidence interval, *VAS* Visual Analog Scale Orofacial, *PPTs* Pressure Pain thresholds, *DTR* distal temporal right, *DTL* distal temporal left, *PTR* proximal temporal right, *PTL* proximal temporal left, *MDR* masseter distal right, *MDL* masseter distal left, *MPR* masseter proximal right, *MPL* masseter proximal left, *TMR* temporomandibular joint right, *TML* temporomandibular joint left, *CF-PDI* Craniofacial Pain and Disability Inventory total, *CF-PDI pain* Craniofacial Pain and Disability Inventory functional pain and disability, *CF-PDI functional* Craniofacial Pain and Disability Inventory functional status of the jaw

^a^Significance level *p* < 0.05

Table 1 shows the participants’ demographic and clinical data. A total of 46 women are diagnosed with FMS (mean age 56.45; 6.93 years), and 44 healthy women (mean age 54.95; 8.11 years) met the inclusion criteria. There were no significant differences in age, weight, height, BMI, tympanic temperature, or skin facial thermography variables. There were significant differences in axillary temperature (*p* ≤ 0.005); status of menopause (*p* ≤ 0.005); and intake of medications such as analgesics, anti-inflammatory drugs, anxiolytics, and antidepressants (*p* ≤ 0.005). Regarding clinical data, there were significant differences in VAS orofacial pain (*p* ≤ 0.001), all orofacial PPTs (*p* ≤ 0.001), mouth opening (*p* = 0.003), CF-PDI-total (*p* ≤ 0.001), CF-PDI-pain (*p* ≤ 0.001), and CF-PDI-jaw-functional (*p* ≤ 0.001) between participants with FMS and healthy controls.

### Association between facial thermography and oral symptomatology

The association between DT temperature and oral symptomatology, including facial pain intensity, facial PPTs, mouth opening, and QoL orofacial, after adjusting by age, BMI, and menopause status in women with FMS, is shown in Table 2. Linear regression analysis revealed an association between DT temperature and MPL (*β* = − 0.363; 95% CI − 0.615, 0.720, *p* = 0.041). Regarding TMJ temperature, significant associations between MDR (*β* = − 0424; 95% CI − 1.425, − 0.131: *p* = 0.020) and MPR (*β* = − 0.424; 95% CI − 1.425, − 0.131: *p* = 0.020) were observed after adjusting for covariates (Table 3). No significant association was found between PM temperature and oral symptomatology (Table 4)*.* Finally, linear regression analysis between DM temperature and oral symptomatology showed significant associations for CF-PDI-total (*β* = 0.415; 95% CI 0.000, 0.002: *p* = 0.015), CF-PDI-pain (*β* = 0.394; 95% CI 0.000, 0.002: *p* = 0.024), and CF-PDI-jaw-functional (*β* = 0.350; 95% CI 0.000, 0.004; *p* = 0.039) (Table 5).

## Discussion

The present study examines the prevalence of orofacial pain in women with FMS compared to healthy women, as well as its potential correlation with the peripheral temperature of facial skin in women with FMS. Our results indicate significant differences in the presence of chronic orofacial pain as assessed by the VAS between women with FMS and healthy women. Additionally, participants with FMS exhibited lower orofacial pain thresholds compared to healthy women, further supporting the higher prevalence of orofacial pain in this patient population. Women with FMS also demonstrated restricted mouth openings relative to controls, suggesting a limitation in the range of mandibular movement. Regarding orofacial QoL, we found that levels of CF-PDI were significantly higher in women with FMS. On the other hand, our results support a lack of alteration in the facial microcirculation in women with FMS since we did not identify significant differences in the peripheral temperature of the facial skin between cases and controls. However, it should be noted that for certain facial thermography variables, we observed significant associations with oral symptomatology.

Chronic pain is defined as recurrent pain over time, and in FMS patients, it has been proposed to be due to the participation of neuronal overestimation and decreased modulation of conditioned pain [10]. Adams et al. [28] indicated that any change in the CNS can cause pain and show a lesion at the somatosensory level. In this state of hyperexcitability, when nociceptive input persists, it leads to increased excitability in spinal cord dorsal horn neurons, resulting in increased responsiveness to noxious and even innocuous stimuli [28, 29]. According to our data, recent studies have also reported the presence of COP in patients with FMS and its impact on the QoL these patients [28]. Sahbaz et al. [29] compared the frequency of TMD and clinical signs along with functionality between women participants with FMS and healthy volunteers and found that bruxism, teeth grinding, and masseter hypertrophy were significantly higher in patients with FMS. Interestingly, Rhodus et al. [30] show a high prevalence of oral symptoms such as xerostomia, glossodynia, or dysphagia. Similarly, a review found that 71–94% of patients with FMS have TMD, a group of musculoskeletal and neuromuscular conditions that affect the masticatory muscles [17]. Thus, it has been shown that a limitation of the range of mouth opening may lead to a limitation in feeding due to a masticatory muscle deficit, generally accompanied by symptoms such as dizziness, tension, tinnitus, and headache [9]. It was also observed that patients with FMS have a lower mechanosensitive threshold to pressure compared to the control group. Since in patients with FMS it has been observed a high prevalence of COP, it has been postulated that FMS could play a relevant role in the chronicity of joint disorders, chronic arthritic conditions, and inflammation of orofacial tissues [8, 9]. Thus, patients with masticatory dysfunctions may show inconsistent pain thresholds during pressure algometry [10]. Based on our data and previous evidence, a complementary orofacial evaluation that includes the PPT evaluation could be an effective tool to determine the orofacial diagnosis in patients with FMS [17]. In addition, it has been observed that in patients with FMS, there is an increase in the concentration of erythrocytes and a decrease in the speed of circulation, which would be related to a decrease in temperature in the regions of the PPTs [27]. Therefore, the management of FMS may include an evaluation of orofacial pain to determine the minimum level of pressure-induced pain and its relationship to hyperalgesia and hyperthermia [10].

In the present study, we report the lack of significant differences in peripheral facial skin temperature between cases and controls. In contrast to our study, Haddad et al. also identified correlations between PPT measurements of myofascial, masseter, and anterior temporal pain syndrome and the presence of altered peripheral microcirculation in the masticatory muscles, supporting the presence of local hypoxia [18, 31]. These preliminary findings support that orofacial thermography might be a reliable tool for the assessment of orofacial pain [18]. Almeida et al. [32] also identified differences in thermal distribution and a muscle abnormality caused by increased tension or pain. Interestingly, in a study conducted in patients with FMS and healthy subjects, Sempere-Rubio et al. [19] showed no significant thermal differences in skin temperature between the groups on the neck or upper back. The inconclusive results may be due to differences in the delineation of the ROI. In this line, Ludwin et al. indicated that ROI areas are reliable but may be limited to maintaining the same area in each participant, which is conditioned by orofacial area and body size [33], and, therefore, there is a need to establish a more accurate method to delineate ROI. Also, to date, no standardized protocols for facial thermography in FMS have been previously establish. Therefore, studies with larger study samples with exhaustive protocols for determining ROI and thermography temperature will be necessary to validate our preliminary findings.

To our knowledge, our study is the first to assess the association between peripheral temperature of the facial skin and orofacial pain assessed by PPTs in patients with FMS. Significant associations were observed between thermography and PPTs for MPL and DT temperature; MDR and MPR and TMJ temperature. An association was also observed between DM temperature and CF-PDI-total, pain and jaw-functional. The findings of this study have clinical implications that are relevant in the management of FMS symptomatology [34]. Chronic orofacial pain may require a multidisciplinary approach, involving nurses, rheumatologists, physiotherapists, and speech therapists, each contributing to patient education and symptom management by providing information about the disease, treatment options, and self-management strategies, including lifestyle modifications, regular exercise, and a balanced diet [2, 34]. Additionally, rheumatologists specializing in early diagnosis and pain management; physiotherapists to design exercise programs tailored to individual capacities; and speech therapists to manage orofacial pain, oral self-care, and oral symptoms such as xerostomia, oropharyngeal dysphagia, or odynophagia would also be required [9, 34]. A thorough evaluation and comprehensive intervention could aid in early diagnosis and rehabilitation, consequently improving the quality of life for patients with FMS. However, our findings should be considered preliminary, and further studies are needed to examine the ROI assessed by thermography, which is required to contribute to the diagnosis of chronic orofacial pain in participants with FMS [8].

This study has several limitations that need to be addressed. Firstly, due to its transversal nature, no casual relationships have been established. Second, our study sample consisted of a population of women with FMS, and therefore our data may not be generalizable to other populations. Third, given the exploratory nature of this research and the limited availability of prior data on microvascular facial blood flow in patients with FMS, the sample size was determined based on feasibility considerations and alignment with similar studies in the field. Thus, the absence of a formal power analysis to determine the sample size is a potential limitation of the present study, and future studies with larger samples and prospective power calculations are warranted. Despite its limitations, the present study still has a number of strengths since this is the first study that examines the associations between the presence of chronic orofacial pain included in the variables VAS, PPTs, mouth opening, CF-PDI, and subscales in participants with FMS. It is also a pioneering study on the association between hyperthermia of certain orofacial areas, evaluated by thermography, and orofacial pain regions.

Women with FMS presented with greater orofacial pain and limited mouth opening, as well as worse orofacial QoL than healthy women. Some associations between facial thermography and oral symptomatology were observed, supporting the possible effect of peripheral facial skin temperature on orofacial pain in women with FMS. Future studies are required to further the impact of facial thermography in FMS and evaluate its potential inclusion in the guidelines for clinical management of FMS.

## Data Availability

All data generated or analyzed during this study are included in this published article. Data from this study are available upon reasonable request to the author.
